# Effective Equine Immunization Protocol for Production of Potent Poly-specific Antisera against *Calloselasma rhodostoma*, *Cryptelytrops albolabris* and *Daboia siamensis*

**DOI:** 10.1371/journal.pntd.0003609

**Published:** 2015-03-16

**Authors:** Sompong Sapsutthipas, Poh Kuan Leong, Surasak Akesowan, Ronachai Pratanaphon, Nget Hong Tan, Kavi Ratanabanangkoon

**Affiliations:** 1 Department of Microbiology, Faculty of Science, Mahidol University, Bangkok, Thailand; 2 Department of Pharmacology, Faculty of Medicine, University of Malaya, Kuala Lumpur, Malaysia; 3 Queen Saovabha Memorial Institute, Bangkok, Thailand; 4 Division of Biotechnology, Faculty of Agro-industry, Chiang Mai University, Chaing Mai, Thailand; 5 Molecular Medicine, Faculty of Medicine, University of Malaya, Kuala Lumpur, Malaysia; 6 Laboratory of Immunology, Chulabhorn Research Institute and Chulabhorn Graduate Institute, Thailand; University of Newcastle, AUSTRALIA

## Abstract

Snake envenomation has been estimated to affect 1.8 million people annually with about 94,000 deaths mostly in poor tropical countries. Specific antivenoms are the only rational and effective therapy for these cases. Efforts are being made to produce effective, affordable and sufficient antivenoms for these victims. The immunization process, which has rarely been described in detail, is one step that needs to be rigorously studied and improved especially with regard to the production of polyspecific antisera. The polyspecific nature of therapeutic antivenom could obviate the need to identify the culprit snake species. The aim of this study was to produce potent polyspecific antisera against 3 medically important vipers of Thailand and its neighboring countries, namely *Cryptelytrops albolabris* "White lipped pit viper" (CA), *Calleoselasma rhodostoma* “Malayan pit viper” (CR), and *Daboia siamensis* “Russell’s viper” (DS). Four horses were immunized with a mixture of the 3 viper venoms using the ‘low dose, low volume multi-site’ immunization protocol. The antisera showed rapid rise in ELISA titers against the 3 venoms and reached plateau at about the 8th week post-immunization. The in vivo neutralization potency (P) of the antisera against *CA*, *CR* and *DS* venoms was 10.40, 2.42 and 0.76 mg/ml, respectively and was much higher than the minimal potency limits set by Queen Soavabha Memorial Institute (QSMI). The corresponding potency values for the QSMI monospecific antisera against *CA*, *CR* and *DS* venoms were 7.28, 3.12 and 1.50 mg/ml, respectively. The polyspecific antisera also effectively neutralized the procoagulant, hemorrhagic, necrotic and nephrotoxic activities of the viper venoms. This effective immunization protocol should be useful in the production of potent polyspecific antisera against snake venoms, and equine antisera against tetanus, diphtheria or rabies.

## Author Summary

Snake envenomation is a serious medical problem in various tropical developing countries. Though antivenoms are the main and rational treatment, they are often of low potency, expensive and/or not available. Moreover, most antivenoms are specific for the treatment of one single snake species and thus necessitate catching the culprit snake so the correct antivenom can be administered. Studies currently are being conducted with the aim of eliminating these shortcomings and to produce polyspecific antivenom capable of neutralizing multiple snake venoms. Production of snake antivenoms involves immunization of an animal, usually horses, with snake venom(s). The serum or plasma is then fractionated to yield antivenom antibodies. While the fractionation processes have been well established, the immunization protocol for the preparation of high potency antisera has rarely been reported in detail. This report describes an effective immunization protocol for the production of potent polyspecific horse antisera against all 3 medically important viper venoms of Thailand (Russell’s viper, Malayan pit viper and green pit viper). The antivenom prepared from the antisera should be useful for the treatment of these viper bites. The immunization protocol should be useful in the production of other potent polyspecific antisera as well as equine antisera against other diseases.

## Introduction

Snake envenomation is an important yet neglected health problem in many poor tropical countries [1,2] with an estimated 1.8 million people are affected worldwide resulting in approximately 94,000 fatalities annually [3]. Antivenoms are considered to be the only rational and effective treatment for envenomation by snakes. In recent years, studies on various research fronts are being conducted to improve the potency and availability of antivenoms [4–6]; it has been suggested that effective immunization to produce potent polyspecific antisera is one important step that needs to be achieved.

In the past, antisera were produced by immunization of horses with snake venom using bentonite as an adjuvant; the potent complete Freund’s adjuvant (CFA) was not used since it causes severe local reactions in horses [7]. Few horses responded to this immunization program and the antisera thus produced were of low potency, leading to shortage of the life-saving antivenoms [8]. In order to produce high potency antivenoms, various toxin/venom immunogens, adjuvants, formulations and immunization schedules for production have been studied [9,10]. It was shown that bentonite, and not the highly toxic venom protein toxins, was the cause of the poor antibody response observed. Pratanaphon et al. [11] showed that if the CFA emulsified immunogen preparation was injected in small volumes (i.e., 0.1–0.2 ml) at many sites covering a wide anatomical area of the neck, severe adverse local reactions caused by the adjuvant could be avoided. This simple immunization protocol has resulted in a dramatic increase in the numbers of responder horses and also in the potency of the antisera [8,11]. The ‘low dose low volume, multi-site’ immunization method has been used successfully in the production of potent polyspecific antisera against 3 elapid venoms [12]. El-Kady et al. [13] showed that the significant improvement in potency achieved by this protocol was due to the production of specific antibodies against the venom toxins/proteins that prossessed high affinity. A low venom dose, complete Freund’s adjuvant and incomplete Freund’s adjuvant (CFA/IFA) immunization protocol was shown to successfully produce potent polyspecific antisera against viperid venoms [14].

Antivenoms against snakes can be monospecific or polyspecific. The selection of a monospecific antivenom for treatment is based on identification of the culprit snake and on the signs and symptoms present in patients after snakebite. When the identification of the envenoming species is not certain, it is better to use a polyspecific antivenom that is effective against venoms of several species of snakes present in the area [14,15]. The aim of the present study was to use a ‘low dose low volume, multi-site’ immunization strategy which had been shown to be highly effective with elapid (cobra and kraits) venoms [11,12], to produce potent polyspecific antisera against the three medically important vipers (*Calloselasma rhodostoma*, *Cryptelytrops albolabris* and *Daboia siamensis*) of Thailand and its neighboring countries while at the same time causing minimal side-effects on the horses. It is reported here that the immunization protocol yielded potent polyspecific antisera against these 3 vipers.

## Materials and Methods

### Chemicals and biochemicals

CFA, IFA, aluminum phosphate and chemicals (reagent grade) were purchased from Sigma Chemical Company, St. Louis, Missouri, USA, except as indicated. The snakes were obtained from various regions throughout Thailand and also from breeding at the snake farm of QSMI, Bangkok. Lyophilized crude venoms from *Calloselasma rhodostoma* “Malayan pit viper”, *Cryptelytrops albolabris* “White lipped pit viper” and *Daboia siamensis* “Russell’s viper” each pooled from dozens of adult specimens milked manually at QSMI. Monospecific antisera against each of these viper venoms were obtained from horses immunized with the venoms, with either CFA/IFA or bentonite as the adjuvant; and the sera were prepared from clotted blood at QSMI. Hemato Polyvalent Snake Antivenom (HPAV) (Lyophilised; Batch no. 0020107; Exp. Date November 06^th^, 2013), a commercial purified F(ab’)_2_ obtained from serum of equines hyperimmunized against a mixture of three venoms: *C*. *rhodostoma* (Malayan pit viper), *C*. *albolabris* (white-lipped pit viper) and *D*. *siamensis* (Russell’s viper), all of Thai origin.

### Animal ethics statement

Experiments involving horses and mice used in *in vivo* lethality neutralization assay were reviewed and approved by the Animal Care and Use Committee of the Faculty of Science, Mahidol University, Protocol no. MUSC56–008–270. The snakes were maintained and milked at the snake farm of the QSMI following the Regulation of the QSMI Safety Committee for Good Practice on Venomous Snakes in accordance with the WHO Guidelines for the Production Control and Regulation of Snake Antivenom Immunoglobulins 2010 (http://www.who.int/bloodproducts/snakeantivenoms). Animal (mice) studies in the neutralization experiments of procoagulant, hemorrhagic, necrotic and nephrotoxic activities of the viper venoms were reviewed and approved by the Animal Care and Use Committee (ACUC) of the University of Malaya (Ethical clearance letter No. 2013–06–07/MOL/R/FSY).

### Preparation of immunogens

A mixture of crude venoms from the three vipers was emulsified in CFA, IFA or aluminum phosphate as described by Pratanaphon et al. [11].

### Immunization of horses

A group of 4 horses weighing 210–370 kg, aged 3–10 years were immunized with the mixture of three crude venoms prepared in the adjuvants. Venom proteins of increasing dose (1–7 mg) were injected subcutaneously at the neck by the low volume, multi-site protocol at biweekly intervals [11,12]. Details on the immunization, including the venom dose, adjuvant, volume, number of sites of injection and schedule are shown in Table 1.

**Table 1:** Detail information on the immunization program: the venom, venom doses, adjuvants and route of injection during the course of immunization.

Week	Immunogen composition	Dose (mg)	Total volume (ml)	Bleeding no.
0	*(CA+CR+DS)+CFA	1+1+1	2 ml (10 sites x 0.2 ml)	1
2	(CA+CR+DS)+IFA	3+3+3	2 ml (10 sites x 0.2 ml)	2
4	(CA+CR+DS)+AlPO_4_	3+3+3	4 ml (4 sites x 1 ml)	3
6	(CA+CR+DS)+AlPO_4_	5+5+5	4 ml (4 sites x 1 ml)	4
8	(CA+CR+DS)+AlPO_4_	7+7+7	4 ml (4 sites x 1 ml)	5
10	-	-	-	6
14	-	-	-	7
18	-	-	-	8
22	CR+IFA	5	2 ml (10 sites x 0.2 ml)	9
24	CR+AlPO_4_	5	4 ml (4 sites x 1 ml)	10
26	-	-	-	11
28	-	-	-	12
32	(CA+CR+DS)+IFA	5+5+5	2 ml (10 sites x 0.2 ml)	13
34	(CA+CR+DS)+AlPO_4_	7+7+7	4 ml (4 sites x 1 ml)	14
36	-	-	-	15

* (CA+CR+DS): mixture of three crude venoms: CA, *C*. *albolabris*; CR, *C*. *rhodostoma;* DS, *D*. *siamensis*.

### General well-being of the horses

Weight and reactions at immunization sites were monitored. Weight was monitored monthly during the immunization course. Reactions at injection sites were recorded by measuring the diameter of the inflammation zone at each site and then calculating the mean. The reaction score at each injection site was graded as previously described by Pratanaphon et al. [11].

### Determination of specific antibody titer in horse sera

The indirect technique of Enzyme Linked Immuno-Sorbent Assay (ELISA) was used to assay the antibody titer of the horse antisera against each viper venom. The assay was carried out as described by Rungsiwongse and Ratanabanangkoon [16] which was based on Theakston and Reid [17] with some modifications. A polyvinyl microtiter plate (Cat. No. 18897005 Costar) was coated with 50 μL/well of 5 μg/ml of each viper venom in 0.05M sodium carbonate–bicarbonate buffer pH 9.6 and incubated for 18 h at 4 C in a moist chamber, followed by washings with normal saline containing 0.05% Tween-20. Serum samples, positive and negative references were diluted serially fivefold from 1:250 to 1:156250 in diluting buffer (0.15M PBS containing 0.05% Tween-20 and 0.5g% BSA). Each sample was assayed in duplicate. A 50 μL amount of each sera dilution was added to each well and incubated for 1 hour at room temperature. After 3 washings, 50 μL of 1:160 diluted sheep anti-horse lgG-Horse radish peroxidase (HRP) conjugate in diluting buffer was added to each well except the conjugate blank well to which only diluting buffer was added. After 1 hour incubation and four washings, each well was supplemented with 100 μL of the substrate solution (40 mg% of a-phenylenediamine and 0.003% hydrogen peroxide in 0.075 M citrate-phosphate buffer pH 5.0). The reaction was stopped by adding 25 μL of 4N H_2_SO_4_ to each well. The absorbance at 492 nm was read against substrate blank using Multiskan MCC/340 MK IT. Antibody titers of sera samples were determined by comparing with a positive reference serum which was also included in every plate to correct for day-to-day and plate-to-plate variations. The corrected mean ELISA OD of each sample was plotted against the reciprocal dilution of the serum. The dilution giving an ELISA OD reading of 0.5 was regarded as the end-point titer.

### Determination of median lethal dose (LD_50_) of the three viper venoms

The method described by Theakston and Reid [18] was used to determine the lethality of each of the venoms. Swiss albino mice weighing 20 + 2 g, supplied by the National Laboratory Animal Center, Mahidol University, were used. Groups of 5 mice were injected intra-peritoneally with *C*. *albolabris* venom at doses ranging from 0.93 to 2.10 μg/g. mouse, with *C*. *rhodostoma* venom at 3.85 to 9.64 μg/g. mouse or with *D*. *siamensis* venom at 0.25 to 1.03 μg/g. mouse. The venoms were prepared in sterile normal saline solution (NSS) and the volume of injection was kept constant at 200 μL/20 g. mouse. Control animals were injected with NSS only. All injections were made using a 250 μL Hamilton syringe. The percent death of animals was recorded 24 hours after injection and the median lethal dose was determined by the probit method [19].

### Determinations of the *in vivo* neutralization potencies of horse Hemato polyspecific antisera (HP antisera) against three viper venoms

Equal volumes of sera from the 15^th^ bleeding of the 4 horses were pooled (termed hemato-polyspecific antisera or HP antisera) and used for the neutralization studies.

The *in vivo* neutralization activity of horse HP antisera was determined in mice according to the method of Theakston and Reid [18] with modifications. Five LD_50_’s of *C*. *albolabris* venom or *D*. *siamensis* venom, or three LD_50_’s of *C*. *rhodostoma* venom were mixed separately with increasing doses of HP antisera (6.25 to 200 μL), then made up to 250 μL in NSS. The venom-antiserum mixture was allowed to stand for 30 minutes at room temperature, after that it was kept on ice until used. The venom-antisera mixture was injected intra-peritoneally into each group of five mice. The volume of the mixture was kept constant at 250 μL (200 μL of serum and 50 μL of venom solution) while control mice were injected intra-peritoneally with normal saline solution (NSS) at 250 μL/20 g. mouse. All injections were made using a 250 μL Hamilton syringe. The number of surviving animals was observed after 24 hours and the median effective dose (ED_50_) was estimated by probit methods [19]. ED_50_, the ratio of venom in mg per ml of HP antisera that gives 50% survival of mice tested, as well as the neutralization potency (P), the amount of venom completely neutralized by one ml of antisera were calculated [20]. The dose was estimated using the formula P = (n—1)LD_50_ / ED_50_where n is the challenge dose.

### Neutralization of pro-coagulant, hemorrhagic, necrotic and nephrotoxic activities

The venom procoagulant activity was assessed according to Theakston and Reid [18], while hemorrhagic and necrotic activities were assessed as described by Gutierrez et al. [21] and Ramos-Cerrillo et al. [15], respectively. The procoagulant activity of the venom in fibrinogen or plasma was expressed as minimal coagulant dose in fibrinogen (MCD-F) or (MCD-P); defined as the venom dose that coagulates the fibrinogen or plasma within 60 seconds under the assay conditions. Minimal hemorrhagic dose, MHD, was defined as the amount of venom that induces a skin hemorrhagic lesion of 10 mm diameter; while the minimal necrotic dose, MND, was defined as the amount of venom that induces a skin necrotic lesion of 5 mm diameter.

Neutralization assays of venom procoagulant, hemorrhagic and necrotic activities by the HP antisera were carried out as described by Leong et al. [22]. The neutralization of hemorrhagic and necrotic activities were expressed as median effective dose (ED_50_), defined as the amount of antisera in μL or the ratio of mg venom/mL antisera in which the venom activity was reduced by 50%. The neutralization of procoagulant activity was expressed as effective dose (ED), defined as the amount of antiserum in μL or the ratio of mg venom/mL HP antisera in which the clotting time was prolonged three times compared to that of fibrinogen or human plasma incubated with venom.

The *in vivo* neutralization of nephrotoxicity of *D siamensis* venom by HP antisera was assessed according to Tan et al. [23]. A control group of mice (20–25 g, n = 3) received intramuscular injection of physiological saline into the thigh muscles. An envenomed group of mice (n = 3) received intramuscular injection of 15 μg, or approximately 1/3 *i*.*m*. LD_50_ (*i*.*m*. LD_50_ = 2.0 μg) of *D siamensis* venom. The treatment group (n = 3) received intravenous injection of 200 μL antisera at 10 minutes after the injection of venom. Urine samples from all groups were collected pre-envenomation and at 4 h post-envenomation using catheter. The urine samples were screened for hematuria and proteinuria, using Roche Combur 10-test M strips (Roche, Germany). Blood was collected from the mice under urethane anesthesia (1.4 mg/g) for urea and creatinine analysis by an independent pathology laboratory service center. Proteinuria was based on the following scale: negative (<10 mg/dL), trace (10 mg/dL), 1+ (30 mg/dL), 2+ (100 mg/dL), 3+ (300 mg/dL), and 4+ (1,000 mg/dL or greater). Hematuria was based on the following scale: negative (< 10 rbcs/μL), trace (~10 rbcs/μL), 1+ (~25 rbcs/μL), 2+ (~50 rbcs/μL), 3+ (~150 rbcs/μL) and 4+ (≥250 rbcs/μL).

### Miscellaneous procedures

Protein concentration was determined by the procedure described by Lowry et al. [24] using bovine serum albumin as the standard. Statistical analysis of the data on horse reactions was made using a t-test to compare severity scores among groups of horses and paired-t-test for scores of CFA and IFA in the same group. LD_50_ and ED_50_ with the 95% confidence intervals were calculated using the probit method of Finney [19].

## Results

### Cross-reactivity of horse antibody against venoms of the 3 vipers

In order to see whether immunochemical cross-reactivity between the venom and antiserum would be a problem in the interpretation of ELISA results of the HP antisera, the ELISAs of homologous and heterologous venoms (*CR*, *CA* and *DS* venoms) were tested with the 3 monospecific antisera i.e., anti-*CR*, anti-*CA* and anti-*DS*. With the monospecific horse antisera, homologous venoms reacted most strongly with titers over 10^4^. Heterologous venoms were shown to cross-react, but at lower titers (10^2^–10^3^) and thus should only slightly interfere with the ELISA results of horse antisera (S1 Fig.).

### Kinetics of horse antibody responses

**Fig 1:**
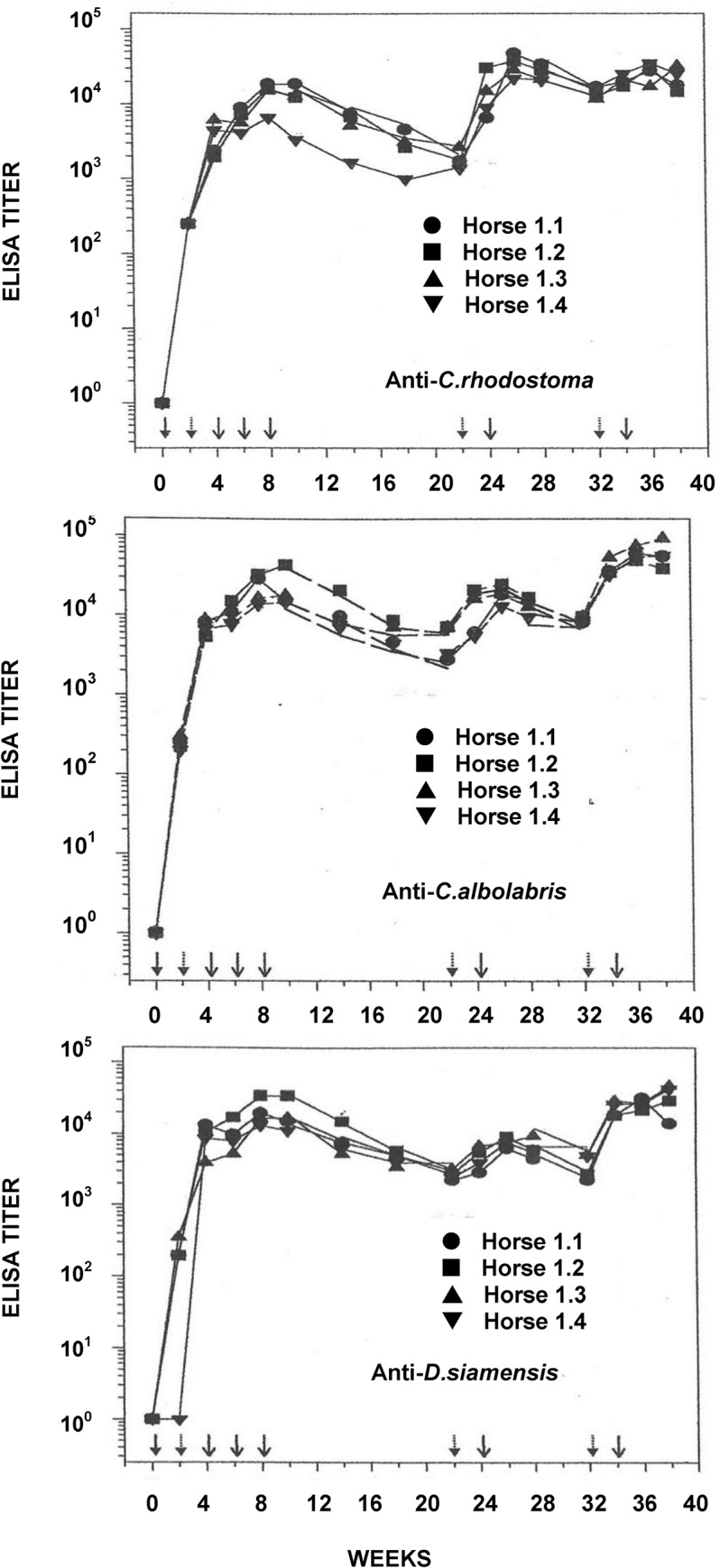
Kinetics of antibody response of horses against *C*. *rhodosoma*, *C*. *albolabris* and *D*. *siamensis* venom. Horses were immunized at biweekly intervals with a mixture of the three viper venoms emulsified with CFA (↓) and then with IFA (⇣) followed by a mixture of the three viper venoms mixed with AlPO_4_ (↓).

The anti-*C*. *rhodostoma* antibody response (Fig. 1) as well as those against *C*. *albolabris* and *D*. *siamensis* venoms rose rapidly and reached plateau at about 8 weeks. With no further immunization, the antibody level slowly declined. At the 22^nd^ week, the anti-*C*. *rhodostoma* ELISA titer, in contrast to those against *C*. *albolabris* and *D*. *siamensis*, fell to very low levels. It was therefore decided that for booster immunization, only *C*. *rhodostoma* venom would be injected. The booster injection resulted in rapid increase in anti-*C*. *rhodostoma* titer within 1–2 weeks. It should be noted that the anti-*C*. *albolabris* and anti-*D*. *siamensis* titers were also increased; this was most likely due to the cross reactions of the heterologous antibodies in the ELISA mentioned earlier. After the final booster round, the antibody titers against all 3 venoms peaked at slightly higher than the previous round. Antibody responses against the all 3 venoms followed similar kinetics. All the horses appeared to respond with high and comparable antibody titers.

### Median lethal doses (LD_50_) of viper venoms

The LD_50_’s of the venoms (with 95% confidence intervals in parentheses) were 1.17 μg/g. mouse (0.83–1.37) for *C*. *albolabris* venom, 5.01 μg/g. mouse (2.34–6.35) for *C*. *rhodostoma* venom and 0.49 μg/g. mouse (0.40–0.60) for *D*. *siamensis* venom (Table 2).

**Table 2:** *In vivo* lethality neutralization potencies of various antisera preparations.

**A. *In vivo* neutralization by polyspecific HP antisera**					
**Venom**	***i*.*p*. LD_50_ (μg/g)**	**Challenge dose**	**Polyspecific HP antisera**		
			**ED_50_ (μl/mouse)**	**ED_50_ in mg/mL**	* **P (mg/mL)**
*Cryptelytrops albolabris*	1.17 (0.83–1.37)	5 LD_50_	9.00	13.00 (9.22–15.22)	10.40
*Calloselasma rhodostoma*	5.01 (2.34–6.35)	3 LD_50_	83.00	3.62 (1.69–4.59)	2.42
*Daboia siamensis*	0.49 (0.40–0.60)	5 LD_50_	51.61	0.95 (0.78–1.16)	0.76
**B. *In vivo* neutralization by monospecific antisera (multisite – CFA immunization)**					
**Venom**	***i*.*p*. LD**_**50**_ **(**μ**g/g)**	**Challenge dose**	**Monospecific antisera**		
			**ED**_**50**_ **(**μ**l/mouse)**	**ED**_**50**_ **in mg/mL**	**P (mg/mL)**
*Cryptelytrops albolabris*	1.17 (0.83–1.37)	5 LD_50_	12.86	9.10 (6.45–10.65)	7.28
*Calloselasma rhodostoma*	5.01 (2.34–6.35)	3 LD_50_	64.16	4.68 (2.19–5.94)	3.12
*Daboia siamensis*	0.49 (0.40–0.60)	5 LD_50_	26.07	1.88 (1.53–2.30)	1.50
**C. *In vivo* neutralization by monospecific antisera (bentonite immunization)**					
**Venom**	***i*.*p*. LD**_**50**_ (μ**g/g**)	**Challenge dose**	**Monospecific antisera**		
			**ED**_**50**_ **(**μ**l/mouse)**	**ED**_**50**_ **in mg/mL**	**P (mg/mL)**
*Cryptelytrops albolabris*	1.17 (0.83–1.37)	5 LD_50_	not available	not available	not available
*Calloselasma rhodostoma*	5.01 (2.34–6.35)	3 LD_50_	>200	<1.5	<1.00
*Daboia siamensis*	0.49 (0.40–0.60)	5 LD_50_	70.71	0.69 (0.57–0.85)	0.56

*P, neutralization potency is the amount of venom completely neutralized by one ml of antisera [20].

2A, polyspecific HP antisera produced by low dose, low volume multi-site CFA immunization; 2B, monospecific antisera produced by low dose, low volume multi-site CFA/IFA immunization; 2C, monospecific antisera produced using bentonite as an adjuvant.

### *In vivo* neutralization potency of horse HP antisera

The horse sera of the 15^th^ bleeding gave the highest ELISA titers and also passed the potency limits of QSMI for the 3 venoms (0.3, 1.2 and 0.3 mg/ml against *C*. *albolabris*, *C*. *rhodostoma* and *D*. *siamensis* venoms, respectively). These sera of the 4 horses were pooled (called HP antisera) and the *in vivo* neutralization potency determined. It should be pointed out that in this determination the maximum volume of the HP antisera used was 200 μL; if this amount failed to neutralize 5LD_50_ of a venom, then 3LD_50_ was used as in the case of *C*. *rhodostoma* venom. The ED_50_ and potency values of the HP antisera against *C*. *albolabris*, *C*. *rhodostoma* and *D*. *siamensis* venoms are shown in Table 2A. For comparison, the results of the 3 monospecific antisera against the viper venoms produced by QSMI using the low dose, low volume multi-site CFA protocol (Table 2B), and by using bentonite as an adjuvant (Table 2C), were also included.

As expected, the HP antisera from the horses were more potent than monospecific antisera produced against *C*. *rhodostoma* and *D*. *siamensis* venoms using bentonite as an adjuvant. Although a monospecific antiserum against *C*. *albolabris* prepared using bentonite as adjuvant was not available for comparison because this adjuvant was no longer used at QSMI, it is likely that the HP antisera would be more potent.

Comparisons were made between neutralizing potencies of HP antisera and monospecific antisera prepared using the same low dose, low volume multi-site immunization protocols. It was found that the HP anti-*C*. *albolabris* potencies gave higher potencies but the anti-*C rhodostoma* and anti-*D*. *siamensis* potencies were lower than those of the monospecific antisera (Table 2A and 2B).

It is interesting that antibody titers, as judged by ELISA, were similar for the three venoms and yet the neutralizing activity against lethality was lower for *D*. *siamensis* venom. This venom had a high lethality, suggesting that it contained neurotoxic components (perhaps neurotoxic phospholipase A_2_s (PLA_2_s); the venom was also devoid of hemorrhagic activity. Since snake venom PLA_2_s are relatively small proteins of about 15 kD [25] and consequently of low immunogenicity, they are usually difficult to be neutralized by the low level of the corresponding antibody in the antisera.

### Neutralization of procoagulant activities of the viper venoms by the HP antisera

Procoagulant activities of *C*. *rhodostoma*, *C*. *albolabris* and *D*. *siamensis* venoms are compared in Table 3. The procoagulant activities in fibrinogen (MCD-F) of the 3 viper venoms varied considerably. While *D*. *siamensis* venom showed no activity, the MCD-F of *C*. *rhodostoma* venom and *C*. *albolabris* venom were 3.25 mg/L and 23.59 mg/L, respectively. However, the 3 venoms exhibited comparable procoagulant activities in plasma (MCD-P: 24–38 mg/L).

**Table 3:** Comparison of the HP antisera (A) and HPAV F(ab’)_2_ (B) in neutralization against procoagulant activities of the three viper venoms.

Venom	^a^Procoagulant activity(mg/L)	^b^Neutralization (mg/mL)
***(A) Neutralization by HP antisera***		
*D*. *siamensis* (Thailand)	MCD-F = No activity	-
	MCD-P = 38.13 ± 3.12	ED = 42.55 ± 5.52
*C*. *rhodostoma* (Thailand)	MCD-F = 3.25 ± 0.64	ED = 10.12 ± 0.57
	MCD-P = 29.63 ± 7.77	ED = 43.11 ± 2.75
*C*. *albolabris* (Thailand)	MCD-F = 23.59 ± 1.34	ED = 27.00 ± 3.40
	MCD-P = 24.04 ± 5.52	ED = 40.15 ± 3.27
***(B) Neutralization by HPAV F(ab’)*_*2*_** ^#^		
*D*. *siamensis* (Thailand)	MCD-F = No activity	-
	MCD-P = 29.7 ± 3.08	ED = 127.13 ± 21.5
*C*. *rhodostoma* (Malaysia)	MCD-F = 12.06 ± 0.33	ED = 7.47 ± 0.02
*C*. *rhodostoma* (Indonesia)	MCD-F = 5.6 ± 0.30	ED = 5.17 ± 0.15
*C*. *albolabris* (China)	MCD-F = 55.3 ± 0.90	ED = 12.90 ± 0.37

^#^Data from Leong et al. (2013).

^a^Pro-coagulant activity is expressed in terms of MCD-F or MCD-P (mg/L), defined as the amount of venom that clots the fibrinogen solution/human plasma in 60 s.

^b^Neutralization was expressed as effective dose (ED), defined as the amount of antivenom in μL or the ratio of mg venom/mL antivenom in which the clotting time was prolonged three times compared to that of fibrinogen or human plasma incubated with venom.

The HP antisera were able to effectively neutralize procoagulant activities of the *C*. *rhodostoma* and *C*. *albolabris* venoms in bovine fibrinogen, with effective dose (ED) of 10 mg/ml and 27 mg/mL, respectively (Table 3). The HP antisera exhibited effective neutralization against procoagulant activities induced by all 3 Thai viper venoms in human plasma (ED >40 mg/mL).

### Neutralization of hemorrhagic activities of selected viper venoms by the HP antisera

The hemorrhagic activities, measured as MHD ± s.d., of *C*. *rhodotosma* and *C*. *albolabris* venoms were 10.8 ± 3.8 and 1.7 ± 0.4 μg/mouse, respectively. *D*. *siamensis* venom did not exhibit hemorrhagic activity. The neutralization of these activities by the HP antisera gave ED_50_ = 10.12 ± 0.6 and 27.00 ± 3.4 mg/mL, respectively (values in ED_50_ ± s.d.)

### Neutralization of necrotic activities of *C*. *rhodostoma* venom by the HP antisera

The Thai *C*. *rhodostoma* venom exhibited necrotic activity with minimal necrotic dose (MND) = 62.8 ± 6.8 μg/mouse (MND ± s.d.). In the neutralization assay, when 2 MND of *C*. *rhodostoma* venom was injected, all the animals died shortly before the development of any necrotic lesion. It was thus not possible to determine the ED_50_ value (dose that causes 50% reduction of necrotic activity). However, when 2 MND of the venom was premixed with 2.5 μL of HP antisera, and the mixture was injected into the animals, all animals survived without development of necrosis, indicating the necrotic activities of the venom were neutralized by the HP antisera.

It should be pointed out that the necrotic activity assay used here was not sensitive enough to measure the necrotic activities of *C*. *albolabris* and *D*. *siamensis* venoms as even at 2 LD_50_ dose, all animal died before necrotic lesion was observed. Hence, neutralization of necrotic activities of these two venoms was not carried out.

### *In vivo* protective action of HP antisera against nephrotoxic effects induced by *D*. *siamensis* venom

The protective actions of HP antisera against nephrotoxic effects induced by *D*. *siamensis* venom were studied. The analysis for proteinuria using urine dipstick showed trace amounts of protein (10 mg/dL), in the urine samples of all the three experimental groups (control, envenomed and HP antisera-treated). The hematuria was not found in the untreated control (< 10 rbcs/μL), but was observed in the *D*. *siamensis*-envenomed group (50–150 rbcs/μL) and was effectively prevented by the HP antisera, as evidenced by the negative hematuria (< 10 rbcs/μL) in antisera-treated group and this was statistically significantly different (*p* <0.005). On the other hand, the blood urea and creatinine levels of the envenomed group did not show significant abnormalities (S1 Table), probably because of the low dose of venom used.

### The general well-being of horses

Monthly horse weight measurements during the immunization course showed no significant weight gain or loss (S2 Table). With respect to reactions at immunization sites, all horses showed only mild reactions and they responded similarly to CFA and IFA adjuvants. No statistically significant differences in reactions induced by the 2 adjuvants were found (S3 Table).

## Discussion

Attempts are being made in various fields of research to produce effective, affordable antivenoms with wide coverage of snake species [4–6]. The fractionation of equine antisera antibody and its fragments have been relatively well worked out [26], and an effective immunization procedure for the production of a polyspecific antivenom against 3 elapids has been established previously [12]. The immunization protocol described here has now shown to successfully produce potent polyspecific antisera against 3 viper venoms. All the horses responded well to the immunization with high ELISA titers at about the 8^th^ week. The HP antisera were shown to effectively neutralize the lethality, procoagulant, hemorrhagic, necrotic and nephrotoxic activities of all or selected homologous viper venoms. Furthermore, the purified Hemato Polyspecific antivenom F(ab’)_2_ (HPAV F(ab’)_2_) product, recently prepared from the present horse HP antisera by QSMI, had also been shown to be highly effective in this regard [22]. The neutralizing potencies of the HPAV F(ab’)_2_ antivenom were generally higher than those of the HP antisera reported here due to the purification and concentration of the F(ab’)_2_ antibody during production processes. Moreover, the pharmacokinetic parameters, for example, the volume of distribution and serum half-life, of the antiserum antitoxin IgG_T_ and of the F(ab’)_2_ in the purified final antivenom product are known to be different [26–28].

The essence of the present immunization protocol is to use low venom dose (1–2 mg of each venom/ horse) and small volume injections (50–100 μL/site) at multiple sites (10–20 sites/horse) using CFA and IFA as adjuvants. The rationale for this protocol is as follows: the low venom dose allows for stimulation of only the high affinity toxin-specific B lymphocytes. The low volume of injection allows for the use of the potent CFA/IFA adjuvants without causing severe local reactions in the horses. Previously, the use of these adjuvants in horses was discouraged since they are highly sensitive to the oil adjuvants [7]. Lastly, the multi-site injection around the lymph node-rich area of the neck allows for the stimulation of more antigen presenting cells by means of increased total surface area of the immunogen droplets. With this immunization protocol, highly potent monospecific antiserum against the Thai cobra (*Naja kaouthia*) was produced [11], followed by the successful production of a potent polyspecific antisera against 3 elapid venoms [12]. It should be mentioned that previously bentonite was used in immunizations for the production of monospecific antivenoms by QSMI. Given the improvements in terms of antisera potency, polyspecificity, the percent responder horses and the shorter time required to achieve hyperimmune state offered by the low dose multisite immunization protocol, bentonite has been replaced by CFA/IFA [8]. This immunization protocol has drastically reduced both the number of horses used in antivenom production and the time required to reach the hyperimmune stage as well as the amount of venoms used for immunization [8]. These improvements, together with the significant improvement in antisera potencies, have increased the antivenom supply produced by QSMI and turned a severe shortage into surplus that allows for export.

From the immunization protocols described by our group [11,12] together with those reported by El-Kady et al [13] and Archundia et al [14], the common features which have led to the production of potent antisera are as follows:

- CFA and IFA were used as adjuvants.
- Very low venom doses of 1–2 mg/horse or less, were used in the primary immunization with CFA.
- The intervals between the injections were 2 weeks.
- Small volume injections at multiple sites were used (Archundia et al [13] used very low doses but did not provide the details on the volume of injection).

These features, if verified by others, could form a general immunization guideline for the production of potent polyspecific antisera. This protocol, together with the studies on other research fronts [4–6], could eventually improve antivenom production and save lives.

In the present study, the kinetics of the antibody response of the horses was similar to that observed previously, i.e. peak antibody titers were reached within the 8^th^ week. This was much faster than that in horses immunized with bentonite adjuvant (16^th^ week) [11] and could significantly reduce the cost of maintaining the horses. In addition, higher antibody titers were reached after additional rounds of booster. All the horses responded well with high antibody titers against each of the 3 viper venoms. Furthermore, when it was observed that the antibody response against one of the venoms (venom of *C*. *rhodostoma*) was lower than anticipated, booster immunization could be made with only that venom to raise the antibody titer. These response characteristics should be beneficial to the production of potent polyspecific antisera since difference in immunogenicities of venoms, and consequently difference in antibody response is to be expected.

*In vivo* neutralization potencies, as measured by ED_50_s of the HP antisera against *C*. *rhodostoma* and *D*. *siamensis* were much higher than those of the respective monospecific antisera produced using bentonite as adjuvant. Moreover, the neutralizing potency (ED_50_) of the HP antisera against *C*. *albolabris* was very high at 13 mg venom/ml serum which was higher than that of the monospecific anti-*C*. *albolabris* prepared using low dose, multi-site CFA immunization (Table 2A and 2B). Thus, it is likely that monospecific anti-*C*. *albolabris* serum prepared using bentonite, if available, would be lower in potency than the polyspecific HP antisera.

The potency of the polyspecific anti-*C*. *albolabris* antisera was higher, while those of the anti-*C*. *rhodostoma* and anti-*D*. *siamensis* were lower than the corresponding values of the respective horse monospecific antisera prepared using similar immunization protocols. These results are not sufficient to conclude whether antigenic competition [29] was a problem in the production of polyspecific antisera. Ramos-Cerrillo et at [15] have shown that effective polyspecific F(ab’)_2_ antivenoms could be produced from immunization of horses with 5 viper or with 6 elapid venoms. Since each snake venom may contain up to a hundred different proteins [30], antigenic competition might become one of the limiting factors when the number of venoms in the immunogen mix is increased beyond a certain point which has not yet been determined.

The *C*. *rhodostoma* and *C*. *albolabris* venoms from Thailand induced coagulation in both human plasma and bovine fibrinogen but *D*. *siamensis* venom was completely devoid of procoagulant activity in fibrinogen solution. This is expected as *D*. *siamensis* has been known to induce coagulation by activating factor V and factor X instead of thrombin-like action [31]. The HP antisera were shown to effectively neutralize the procoagulant activities induced by all viper venoms of Thai origin. Similarly, the hemorrhagic activities of the homologous *C*. *rhodostoma* and *C*. *albolabris* venoms were neutralized effectively by the HP antisera.

Local tissue necrosis is a common clinical feature of pit viper venom envenoming [32]. In the neutralization assay, a dosage of 2.5 μL HP antisera premixed with 2 MND of the venom could effectively prevent the lethal and necrosis-inducing effects of the venom.

In South and Southeast Asia, bites *by Daboia siamensis* are one of the common causes of acute kidney injury (AKI) [33]. The surviving victims may develop chronic kidney disease with consequent heavy medical and economic burden for people in developing countries. In this study, the nephrotoxic effect of *D*. *siamensis* venom was manifested by hematuria. The HP antisera were effective in neutralizing the nephrotoxicity of the homologous Thai *D*. *siamensis* venom.

An epidemiological study carried out by the Ministry of Public Health of Thailand has revealed that culprit snakes were not identified in 75–80% of the snakebite cases in Thailand [34]. This is not surprising since the bites mostly occur at night or in bushy areas. Even when the culprit snake was seen, the information provided by the victim is often not accurate enough for species diagnosis [35]. Of the 24% where species diagnosis was made, most (91.6%) were vipers (i.e. *C*. *albolabris*, *C*. *rhodostoma* and *D*. *siamensis*) [34]. This information indicates that, in the absence of the culprit snake or error in species diagnosis, the use of monospecific antivenom could result in treatment failure and wastage of valuable antivenom. Thus, a polyspecific antivenom which could neutralize all the venoms of snakes in the localities would be very useful for the treatment of snake envenomation. Not only a polyspecific antivenom offers better chance of successful treatment but its production would be less costly. At the QSMI, 7 monovalent antivenoms are produced which required 7 groups of horses and the antisera obtained require separate fractionation processes. These together with the handling, packaging, quality control and inventory keeping, all add to the cost of production. With the success achieved by the production of polyspecific antisera against 3 elapid venoms [12] and against 3 viper venoms reported here, QSMI is now producing 2 polyspecific antivenoms, one against elapid and another against viper venoms which will eventually replace the monospecific antivenoms. However, there is still some concern as to the relative efficacy and the propensity to cause adverse reactions of polyspecific as compared to monospecific antivenoms [36]. Immunochemical and biochemical comparisons of equine polyspecific and monospecific antisera prepared under the same immunization protocols were made [37]. Both antisera showed similar serum protein profiles, comparable amounts of total antitoxin IgG_T_, comparable *in vivo* neutralization activities and apparent dissociation constant (Kd) of anti-toxin antibody. In this regards, Archundia et al [14] found the neutralization potencies for viper venoms were higher with the pentaspecific than with the trispecific horse antisera.

It is interesting and significant that the commercial HPAV F(ab’)_2_ purified from the horse HP antisera was shown to exhibit high degree of paraspecificity against the toxicities of diverse heterologous viper venoms [22], as were also shown by Archundia et at [14] and Ramos-Cerrilo et al [15] in their polyspecific antisera/antivenom. These observations may be due to the conserved structure of the venom toxins renders cross reactivity with the toxin specific antibody [38]. Thus, the HPAV F(ab’)_2_ could neutralize the lethality of venoms from *C*. *rhodostoma* (Malaysia and Indonesia), *C*. *purpureomaculatus* (Malaysia), *C*. *albolabris* (China) and *Popeia popeorum* and *Daboia siamensis* (Myanmar) but not the venom of *T*. wagleri [22]. If these observations are confirmed clinically, the antivenom will be useful in saving lives not only in Thailand but also in its neighboring countries.

In conclusion, the present study has demonstrated the successful production of potent truly polyspecific antisera against 3 vipers using the low dose, low volume multi-site immunization protocol. The commercial polyspecific antivenom in the form of F(ab’)_2_ has now been produced from the HP antisera by QSMI. This potent antivenom should help save the lives of many victims of envenomation by the 3 and other related vipers in many SE Asian countries. The information obtained from this study should be useful for the production of pan-specific antisera against vipers from a wide geographic area. The immunization protocol reported here can be readily applied to the production of other equine therapeutic antisera, including those for tetanus, diphtheria and rabies.

## Supporting Information

S1 Table The nephrotoxic effects induced by *D*. *siamensis* venom as examined by blood urea and creatinine analysis. (DOCX)

S2 Table Horse weight during immunization. (DOCX)

S3 Table Total scores of local reactions at injection sites after immunization with CFA or IFA as adjuvant. (DOCX)

S1 Fig A) The cross-reactivity of various monospecific antisera against *C*.*albolabris* venom. Plates were coated with 50 μl of *C*. *albolabris* venom at a protein concentration of 5 μg/ ml. B) The cross-reactivity of various monospecific antisera againt *C*. *rhodostoma* venom. Plates were coated with 50 μl of *C*. *rhodostoma* venom at a protein concentration of 5 μg/ ml. C) The cross-reactivity of various monospecific antisera against *D*. *russelli* venom. Plates were coated with 50 μl of *D*. *russelli* venom at a protein concentration of 5 μg/ml. The monovalent antisera were diluted 5 fold from 1:250 to 1:156,250. Enzyme conjugate used was at 1:160 dilution.
(TIF)
