# Supplementary material for: Effective Equine Immunization Protocol for Production of Potent Poly-specific Antisera against Calloselasma rhodostoma, Cryptelytrops albolabris and Daboia siamensis
Source: PLoS Negl Trop Dis. 2015 Mar 16;9(3):e0003609. doi: 10.1371/journal.pntd.0003609 (PMC4361046; doi:10.1371/journal.pntd.0003609)
Supplement: S1 Table — (DOCX) [file pntd.0003609.s001.docx]

**S1 Table.** The nephrotoxic effects induced by *D. siamensis* venom as examined by blood urea and creatinine analysis.

|  | **Urea (mmo/L)** | **Creatinine ((μmol/L)** |
| --- | --- | --- |
| Control (n=3) | 15.9 ± 3.3 | 30.0 ± 1.7 |
| Envenomed (n=3) | 10.1 ± 2.1 | 24.7 ± 10.0 |

The blood urea and creatinine levels of the envenomed group did not show significant abnormalities (*p*>0.05)
