# Supplementary material for: Effective Equine Immunization Protocol for Production of Potent Poly-specific Antisera against Calloselasma rhodostoma, Cryptelytrops albolabris and Daboia siamensis
Source: PLoS Negl Trop Dis. 2015 Mar 16;9(3):e0003609. doi: 10.1371/journal.pntd.0003609 (PMC4361046; doi:10.1371/journal.pntd.0003609)
Supplement: S2 Table — (DOCX) [file pntd.0003609.s002.docx]

**S2 Table.** Horse weight during immunization.

| **Month** | **Weight (Kg) of Horse** | | | |
| --- | --- | --- | --- | --- |
|  | **Horse 1.1** | **Horse 1.2** | **Horse 1.3** | **Horse 1.4** |
| 0 | 290 | 256 | 250 | 290 |
| 1 | 283 | 255 | 244 | 295 |
| 2 | 287 | 255 | 253 | 295 |
| 3 | 295 | 255 | 270 | 295 |
| 4 | 290 | 256 | 265 | 303 |
| 5 | 293 | 257 | 287 | 286 |
| 6 | 287 | 252 | 260 | 287 |
| 7 | 295 | 254 | 255 | 291 |
| 8 | 296 | 252 | 255 | 290 |
| 9 | 295 | 254 | 260 | 291 |
| 10 | 293 | 255 | 265 | 290 |
| 11 | 293 | 256 | 265 | 291 |

Horses weight was monitored monthly during the immunization course.
