# Supplementary material for: Effective Equine Immunization Protocol for Production of Potent Poly-specific Antisera against Calloselasma rhodostoma, Cryptelytrops albolabris and Daboia siamensis
Source: PLoS Negl Trop Dis. 2015 Mar 16;9(3):e0003609. doi: 10.1371/journal.pntd.0003609 (PMC4361046; doi:10.1371/journal.pntd.0003609)
Supplement: S3 Table — (DOCX) [file pntd.0003609.s003.docx]

**S3 Table.** Total scores of local reactions at injection sites after immunization with CFA or IFA as adjuvant.

| **Horse number** | **Time after injection (weeks)** | | | | | | | | | | | | | | | |
| --- | --- | --- | --- | --- | --- | --- | --- | --- | --- | --- | --- | --- | --- | --- | --- | --- |
|  | **CFA** | | | | | | | | **IFA** | | | | | | | |
|  | **2** | **4** | **6** | **8** | **10** | **18** | **24** | **28** | **2** | **4** | **6** | **8** | **10** | **18** | **24** | **28** |
| 1.1 | 2 | 4 | 4 | 0 | 5 | 5 | 6 | 8 | 0 | 0 | 6 | 2 | 5 | 4 | 4 | 3 |
|  |  |  |  |  |  |  |  |  |  |  |  |  |  |  |  |  |
| 1.2 | 1 | 11 | 15 | 9 | 11 | 7 | 9 | 11 | 0 | 0 | 21 | 14 | 13 | 2 | 9 | 3 |
|  |  |  |  |  |  |  |  |  |  |  |  |  |  |  |  |  |
| 1.3 | 10 | 3 | 3 | 2 | 0 | 2 | 2 | 1 | 0 | 13 | 0 | 1 | 1 | 1 | 5 | 2 |
|  |  |  |  |  |  |  |  |  |  |  |  |  |  |  |  |  |
| 1.4 | 16 | 3 | 7 | 5 | 15 | 4 | 3 | 6 | 0 | 17 | 5 | 4 | 0 | 2 | 7 | 2 |
| mean | 7.25 | 5.25 | 7.25 | 4.00 | 7.75 | 4.50 | 5.00 | 6.50 | 0 | 7.50 | 8.00 | 5.25 | 4.75 | 2.25 | 6.25 | 2.50 |
| ±SD | 7.90 | 3.86 | 5.44 | 3.92 | 6.60 | 2.08 | 3.16 | 4.20 | 0 | 8.81 | 9.06 | 5.97 | 5.91 | 1.26 | 2.22 | 0.58 |

Reactions at the sites of injection were recorded by measuring the average diameters of each site. The reaction score of each injection site was graded as follows:

0 normal, little selling (< 1 cm), healed wound

1+ 1-2 cm, swelling with or without open wound

2+ 2-3 cm, swelling without open wound

3+ 2-3 cm, swelling with open wound

4+ 3-4 cm, swelling with or without open wound

Since the total injection sites of each immunization of a horse were 10, the maximum reaction score of each horse was 40. The total reaction score of each horse was graded as follow:

0-9 normal / mild reaction

10-19 moderate reaction

≥20 Severe reaction
