# Supplementary figures and images for: Effective Equine Immunization Protocol for Production of Potent Poly-specific Antisera against Calloselasma rhodostoma, Cryptelytrops albolabris and Daboia siamensis
Source: PLoS Negl Trop Dis. 2015 Mar 16;9(3):e0003609. doi: 10.1371/journal.pntd.0003609 (PMC4361046; doi:10.1371/journal.pntd.0003609)

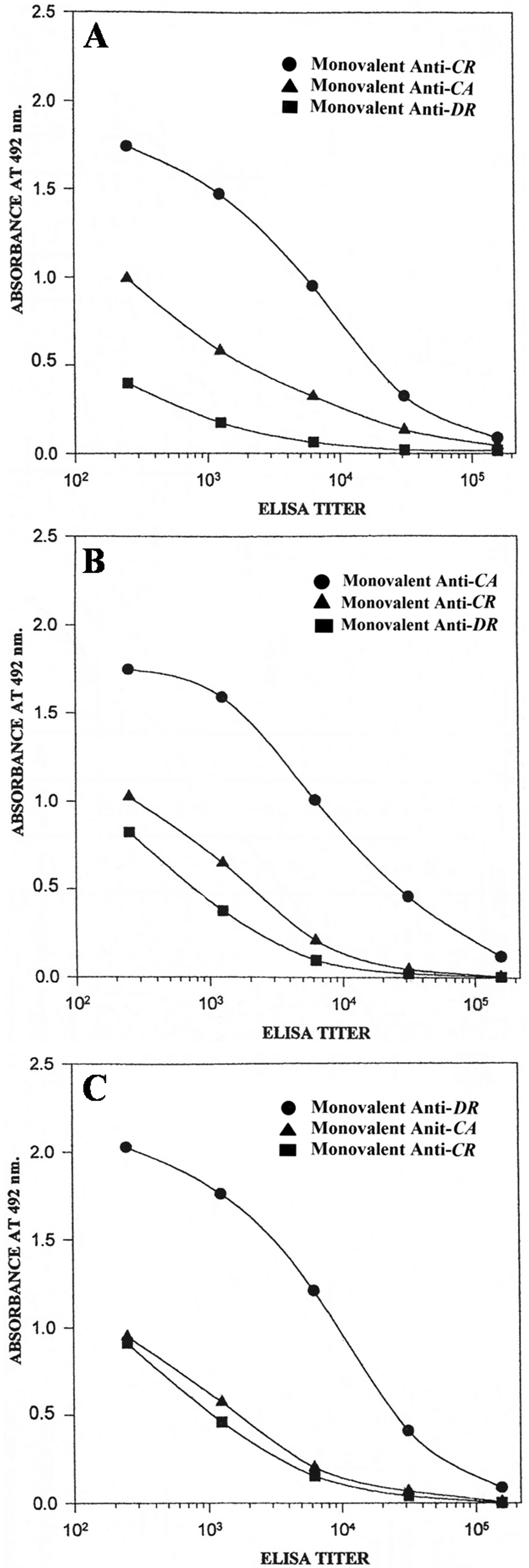

Supplement: S1 Fig — Plates were coated with 50 μl of C. albolabris venom at a protein concentration of 5 μg/ ml. B) The cross-reactivity of various monospecific antisera againt C. rhodostoma venom. Plates were coated with 50 μl of C. rhodostoma venom at a protein concentration of 5 μg/ ml. C) The cross-reactivity of various monospecific antisera against D. russelli venom. Plates were coated with 50 μl of D. russelli venom at a protein concentration of 5 μg/ml. The monovalent antisera were diluted 5 fold from 1:250 to 1:156,250. Enzyme conjugate used was at 1:160 dilution. (TIF) [file pntd.0003609.s004.tif]
